# Partial charge transfer in the salt co-crystal of l-ascorbic acid and 4,4′-bi­pyridine

**DOI:** 10.1107/S2056989019005334

**Published:** 2019-05-03

**Authors:** Eric Sylvester, Mitchell McGovern, An Young Lee, Phanxico Nguyen, Jungeun Park, Jason B. Benedict

**Affiliations:** a 730 Natural Sciences Complex, Buffalo, 14260-3000, USA; b 771 Natural Sciences Complex, Buffalo, 14260-3000, ., USA

**Keywords:** crystal structure, co-crystal, charge transfer, l-ascorbic acid, 4,4′-bi­pyridine

## Abstract

In the title 1:2 co-crystal, C_10_H_9_N_2_^+^·(C_6_H_7.75_O_6_·C_6_H_7.25_O_6_)^−^, l-ascorbic acid (LAA) and 4,4′-bi­pyridine (BPy) co-crystallize in the chiral space group *P*2_1_ with two mol­ecules of LAA, and one mol­ecule of bpy in the asymmetric unit. The structure was modeled in two parts due to possible proton transfer from LAA to the corresponding side of the bpy mol­ecule having an occupancy of approximately 0.25 and part 2 with an occupancy of approximately 0.75. In this structure, LAA forms hydrogen bonds with neighboring LAA mol­ecules, forming extended sheets of LAA mol­ecules which are bridged by bpy mol­ecules. A comparison to a related and previously published co-crystal of LAA and 3-bromo-4-pyridone is presented.

## Abstract 2

In the title 1:2 co-crystal, l-ascorbic acid (LAA) and 4,4′-bi­pyridine (bpy) co-crystallize with two mol­ecules of LAA, and one mol­ecule of bpy in the asymmetric unit. The structure was modeled in two parts due to possible proton transfer from LAA to the corresponding side of the bpy mol­ecule having an occupancy of approximately 0.25 and part 2 with an occupancy of approximately 0.75.

## Chemical context   

l-Ascorbic acid (LAA) is an anti­oxidant and integral vitamin, vitamin C, for many biological systems (Frei *et al.*, 1989 ▸; Yogeswaran *et al.*, 2007 ▸). Since humans cannot synthesize LAA naturally, vitamin C is often obtained from digesting fruits and vegetables, including citrus fruits, tomatoes and potatoes (Medicine, 2000 ▸; Yu *et al.*, 2016 ▸). Vitamin C is also produced through the ingestion of dietary supplements composed of LAA or many other ascorbate-containing derivatives including calcium ascorbate, de­hydro­ascorbate, and calcium threonate (Johnston *et al.*, 1994 ▸).
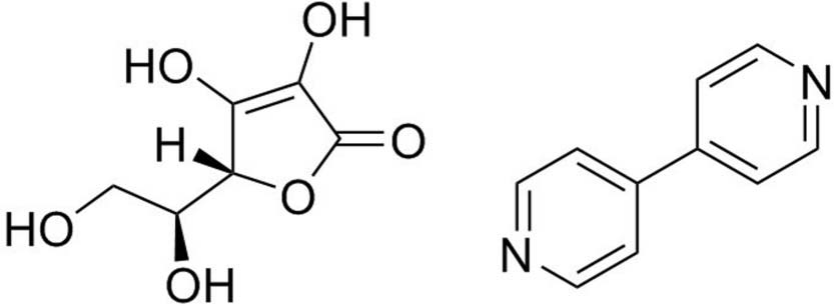


Co-crystallization, a process in which two or more mol­ecules form a crystalline single phase material generally in a stoichiometric ratio (Trask, 2007 ▸), can tailor pharmaceutically important physical properties including solubility, hygroscopicity, and, active lifetime without altering the active pharmaceutical ingredient (Rodriquez-Honedo *et al.*, 2007 ▸; Ross *et al.*, 2016 ▸; Shan & Zaworotko, 2008 ▸; Thipparaboina *et al.*, 2016 ▸). Co-crystal structures are key to identifying important structure-directing inter­actions in the solid-state (Childs *et al.*, 2007 ▸). In this paper, we report the synthesis and single crystal structure determination of a salt co-crystal containing LAA and a commonly used co-former, 4,4′-bi­pyridine (BPy) (Aakeröy *et al.*, 2015 ▸, Cherukuvada *et al.*, 2016 ▸), which is known to be a secondary building component often used as a pillaring ligand to give three-dimensionality in what would normally be stacking of two-dimensional sheets in crystalline systems (Dinesh *et al.*, 2015 ▸; López-Cabrelles *et al.*, 2015 ▸).

## Structural commentary   

**Figure 1:**
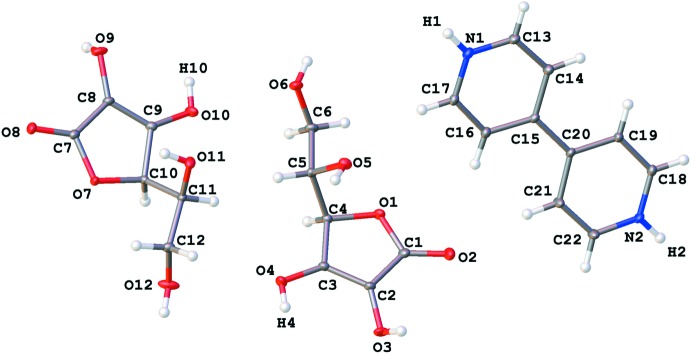
Asymmetric unit of the title compound, showing the numbering scheme.

LAA and BPy co-crystallize in the chiral space group *P*2_1_ with two mol­ecules of LAA, and one mol­ecule of BPy in the asymmetric unit (Fig. 1 ▸). While the lattice is composed of mol­ecules in a variety of charge states (*vide infra*), the neutral mol­ecule abbreviations (LAA and BPy) provide a convenient method for describing the structure in terms of these fragments.

**Figure 2:**
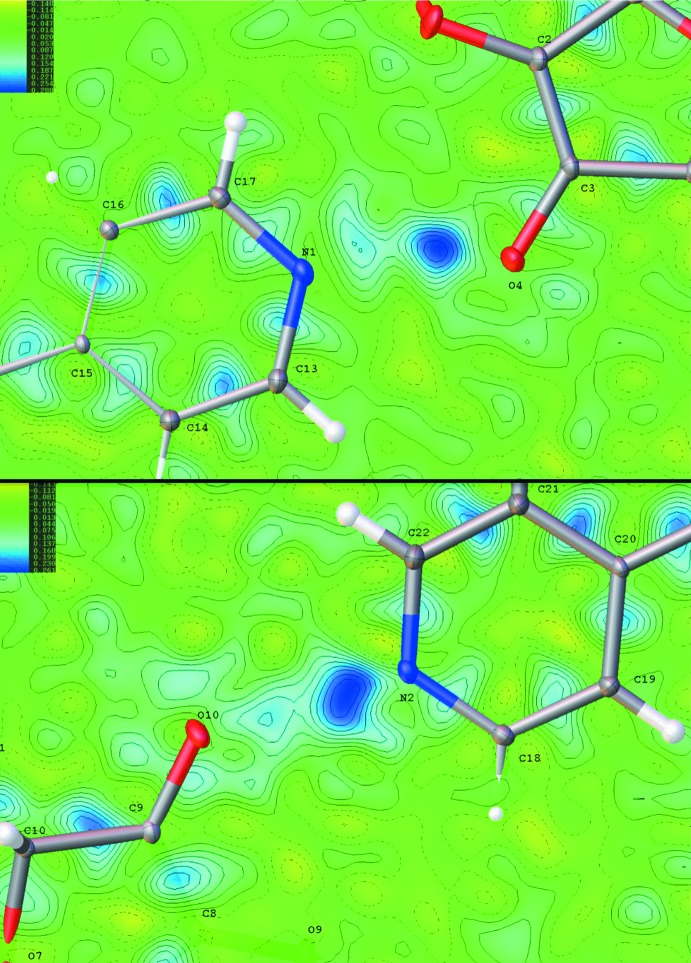
Fourier difference map of the LAA–BPy salt co-crystal showing two peaks of electron density between N1⋯O4 (upper) and N2⋯O10 (lower).

The overall three-dimensional structure is formed by inter­locking sheets of LAA bridged by BPy mol­ecules. Initial attempts to refine the structure as neutral mol­ecules were not satisfactory and suggested the presence of disorder in the positions of the protons involved in inter­molecular hydrogen bonding between LAA and Bpy (H4 and H10). Fourier difference maps produced following a refinement using all atoms except the suspected disorders protons (H4, H10) revealed the presence of two peaks of electron density between the two pairs of heavy atoms involved in the hydrogen bonding (N1 and O4; N2 and O10, Fig. 2 ▸). The positions of the two protons were initially modeled independently (model 1) in two parts to account for the disorder arising from proton transfer from LAA to Bpy. In this model, the occupancy of H10 and its disorder partner atom H2 refined to 0.22736 and 0.70972, respectively. The occupancy of H4 and its disorder partner atom H1 refined to 0.70972 and 0.23932, respectively. The similarity of the occupancies for the two pairs indicated that the disorder was likely correlated.

An additional refinement was performed in which the occupancies were constrained to be identical for the pairs of atoms (single part command for both pairs, model 2). The occupancies for model 2 were determined to be 0.73718 and 0.26282 for the pairs, similar to what was observed in model 1. The *R*_1_ values for both model 1 and model 2 were found to be 3.94%. Given the same values for *R*_1_ for both models, the model with the fewer parameters, model 2, will be reported. There has been an active debate in the community whether an organic salt due to proton transfer is considered a co-crystal (Aakeröy *et al.*, 2007 ▸; Cruz-Cabeza, 2012 ▸; Wang *et al.*, 2018 ▸). However, as we cannot rule out the presence of a non-ionized species within the lattice, we will refer to the obtained product as a salt co-crystal (Cherukuvada *et al.*, 2016 ▸).

## Supra­molecular features   

**Figure 3:**
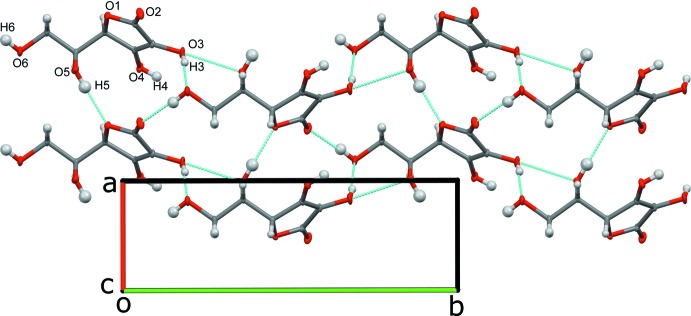
Diagram illustrating the hydrogen-bonding inter­actions (dashed lines, see Table 1 ▸) present in the two-dimensional sheets of LAA mol­ecules, looking down [001]; BPy inter­actions were omitted for clarity.

**Figure 4:**
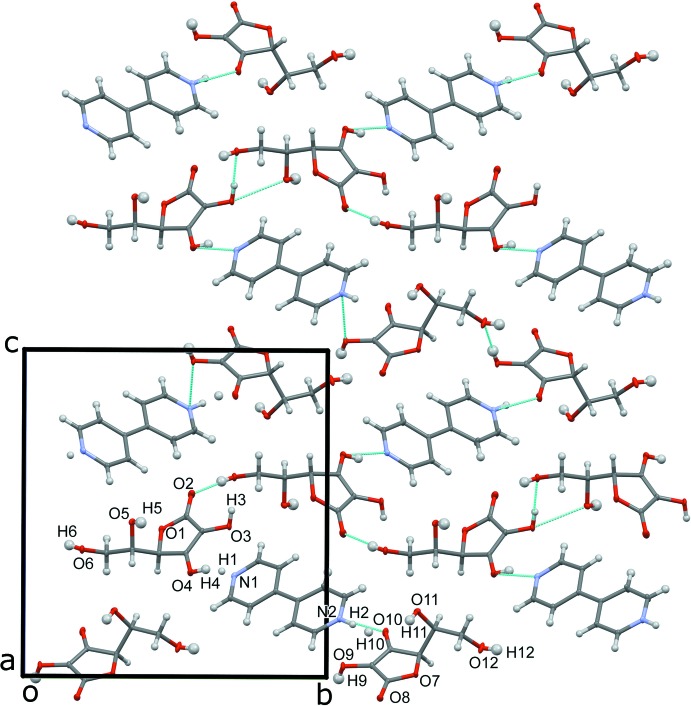
View down [100] showing the packing of the title compound.

In the structure, LAA forms hydrogen bonds with neighboring LAA mol­ecules, giving rise to extended sheets of LAA mol­ecules which are bridged by BPy mol­ecules (Table 1 ▸, Fig. 3 ▸). The LAA–LAA inter­actions consist of O—H⋯O—H hydrogen bonds where each LAA forms a total of three hydrogen bonds with three different LAA mol­ecules, O—H⋯O=hydrogen bond where each LAA forms a hydrogen bond with one different LAA, and O—H⋯O_ether_ where each LAA forms a hydrogen bond with one different LAA. The LAA–BPy inter­action consists of O—H⋯N_pyrid­yl_ hydrogen bonds such that each BPy forms a hydrogen bond with two neighboring LAA mol­ecules (Fig. 4 ▸). C—H⋯O inter­actions also occur.

## Database survey   

**Figure 5:**
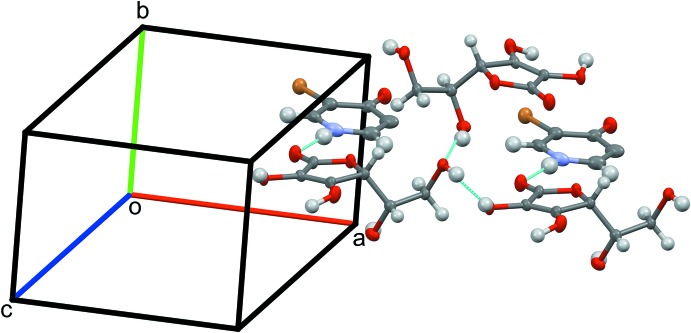
Diagram illustrating the hydrogen-bonding network of the previously reported structure of LAA–BPyBr (Wang *et al.*, 2016 ▸).

Recently the co-crystal structure of LAA and 3-bromo-4-pyridone (BrPyd) was reported (Wang *et al.*, 2016 ▸). While the LAA mol­ecules in each structure contain similar inter­actions, LAA–BPy and LAA–BrPyd demonstrate important differences with regard to the three-dimensional structure because of the different binding synthons of BrPyd compared to BPy (Fig. 5 ▸). In the structure of LAA–BrPyd, the carbonyl on the BrPyd hydrogen bonds with both hydroxyl groups located on the five-membered ring of LAA, whereas the carbonyl located on the five-membered ring of LAA hydrogen bonds with the pyridinium group of BrPyd. The corresponding hydrogen-bond network results in two-dimensional sheets. The three-dimensional aspect of LAA–BrPyd arises from stacking of the sheets, which are held together by hydrogen bonding of the terminal hydroxyl group of the aliphatic carbon chain with the hydroxyl group on the five-membered ring on the LAA in the adjacent sheet.

## Synthesis and crystallization   

All chemicals were obtained commercially and used as received. Solid l-ascorbic acid (0.0450 g, 0.256 mmol) and 4,4′-bi­pyridine (0.0200 g, 0.128 mmol) were added to a 25 ml scintillation vial. To this were added approximately 12 ml of 200 proof ethanol followed by gentle heating. The loosely capped vial was then placed into a dark cabinet. Plate crystals of the title compound suitable for single crystal X-ray diffraction measurements were obtained.

## Refinement   

Crystal data, data collection and structure refinement details are summarized in Table 2 ▸. All H atoms were located in a difference-Fourier map and freely refined. As the Flack parameter is 0.4, the absolute configuration of LAA cannot be determined by the crystal structure; however, the co-crystal was synthesized using an enanti­omerically pure starting material.

## Supplementary Material

Crystal structure: contains datablock(s) I. DOI: 10.1107/S2056989019005334/eb2018sup1.cif

Structure factors: contains datablock(s) I. DOI: 10.1107/S2056989019005334/eb2018Isup2.hkl

CCDC reference: 1910963

Additional supporting information:  crystallographic information; 3D view; checkCIF report

## Figures and Tables

**Table 1:** Hydrogen-bond geometry (Å, °)

*D*—H⋯*A*	*D*—H	H⋯*A*	*D*⋯*A*	*D*—H⋯*A*
N2—H2⋯O10^i^	0.86 (3)	1.74 (3)	2.5862 (14)	169 (2)
O3—H3⋯O5^ii^	0.817 (19)	2.531 (19)	2.8832 (13)	107.5 (15)
O3—H3⋯O6^ii^	0.817 (19)	1.903 (19)	2.7117 (14)	170.0 (19)
O4—H4⋯N1^ii^	0.93 (3)	1.64 (3)	2.5428 (14)	163 (3)
O5—H5⋯O1^iii^	0.85 (2)	2.00 (2)	2.8510 (13)	173 (2)
O6—H6⋯O2^iv^	0.83 (2)	1.84 (2)	2.6616 (14)	173 (2)
O9—H9⋯O12^v^	0.79 (2)	1.91 (2)	2.6902 (14)	175 (2)
O11—H11⋯O10^iii^	0.79 (2)	1.91 (2)	2.6663 (13)	162 (2)
O12—H12⋯O8^vi^	0.87 (2)	1.81 (2)	2.6683 (14)	169 (2)
C5—H5A⋯O11	1.00 (1)	2.44 (1)	3.2950 (14)	143 (1)
C12—H12B⋯O4	0.99 (1)	2.50 (1)	3.3249 (16)	141 (1)
C14—H14⋯O8^vii^	0.95 (1)	2.40 (1)	3.3311 (15)	166 (1)
C16—H16⋯O2	0.95 (1)	2.51 (1)	3.4513 (16)	170 (1)
C17—H17⋯O5	0.95 (1)	2.55 (1)	3.4651 (14)	163 (1)
C19—H19⋯O7^vii^	0.95 (1)	2.56 (1)	3.2181 (14)	127 (1)
C19—H19⋯O8^vii^	0.95 (1)	2.48 (1)	3.4267 (15)	174 (1)
C21—H21⋯O2	0.95 (1)	2.40 (1)	3.3418 (17)	173 (1)
C22—H22⋯O6^viii^	0.95 (1)	2.44 (1)	3.1860 (16)	136 (1)

Symmetry codes: (i) 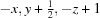; (ii) 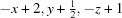; (iii) 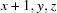; (iv) 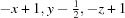; (v) 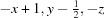; (vi) 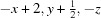; (vii) 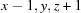; (viii) 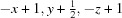.

**Table 2:** Experimental details

Crystal data	
Chemical formula	C_10_H_9_N_2_^+^·(C_6_H_7.75_O_6_·C_6_H_7.25_O_6_)^−^
*M*_r_	508.44
Crystal system, space group	Monoclinic, *P*2_1_
Temperature (K)	90
*a*, *b*, *c* (Å)	4.7724 (6), 14.4069 (17), 15.6857 (19)
β (°)	98.393 (2)
*V* (Å^3^)	1066.9 (2)
*Z*	2
Radiation type	Mo *K*α
μ (mm^−1^)	0.13
Crystal size (mm)	0.2 × 0.1 × 0.02

Data collection	
Diffractometer	Bruker *SMART* APEXII area detector
Absorption correction	Multi-scan (*SADABS*; Bruker, 2013 ▸)
*T*_min_, *T*_max_	0.683, 0.747
No. of measured, independent and observed [*I* ≥ 2u(*I*)] reflections	31611, 9452, 8448
*R*_int_	0.039
(sin θ/λ)_max_ (Å^−1^)	0.809

Refinement	
*R*[*F* ^2^ > 2σ(*F* ^2^)], *wR*(*F* ^2^), *S*	0.040, 0.099, 1.06
No. of reflections	9452
No. of parameters	357
No. of restraints	1
H-atom treatment	All H-atom parameters refined
Δρ_max_, Δρ_min_ (e Å^−3^)	0.47, −0.29
Absolute structure	Flack (1983 ▸)
Absolute structure parameter	0.4 (6)

Computer programs: *APEX2* and *SAINT* (Bruker, 2013 ▸), *SHELXS* (Sheldrick, 2008 ▸), *olex2.refine* (Bourhis *et al.*, 2015 ▸) and *OLEX2* (Dolomanov *et al.*, 2009 ▸).

## supplementary crystallographic information

### Crystal data

**Table:**

C_10_H_9_N_2_^+^·C_6_H_7.75_O_6_0.25^−^·C_6_H_7.25_O_6_0.75−−	*F*(000) = 532.3832
*M**_r_* = 508.44	*D*_x_ = 1.583 Mg m^−^^3^
Monoclinic, *P*2_1_	Mo *K*α radiation, λ = 0.71073 Å
*a* = 4.7724 (6) Å	Cell parameters from 8756 reflections
*b* = 14.4069 (17) Å	θ = 2.6–36.2°
*c* = 15.6857 (19) Å	µ = 0.13 mm^−^^1^
β = 98.393 (2)°	*T* = 90 K
*V* = 1066.9 (2) Å^3^	Plate, yellow
*Z* = 2	0.2 × 0.1 × 0.02 mm

### Data collection

**Table:**

Bruker SMART APEXII area detector diffractometer	9452 independent reflections
Radiation source: microfocus rotating anode, Incoatec Iµs	8448 reflections with *I*≥ 2u(*I*)
Mirror optics monochromator	*R*_int_ = 0.039
Detector resolution: 7.9 pixels mm^-1^	θ_max_ = 35.1°, θ_min_ = 1.9°
ω and φ scans	*h* = −7→7
Absorption correction: multi-scan (SADABS; Bruker, 2013)	*k* = −23→23
*T*_min_ = 0.683, *T*_max_ = 0.747	*l* = −25→25
31611 measured reflections	

### Refinement

**Table:**

Refinement on *F*^2^	37 constraints
Least-squares matrix: full	All H-atom parameters refined
*R*[*F*^2^ > 2σ(*F*^2^)] = 0.040	*w* = 1/[σ^2^(*F*_o_^2^) + (0.0536*P*)^2^ + 0.0945*P*] where *P* = (*F*_o_^2^ + 2*F*_c_^2^)/3
*wR*(*F*^2^) = 0.099	(Δ/σ)_max_ < 0.001
*S* = 1.06	Δρ_max_ = 0.47 e Å^−^^3^
9452 reflections	Δρ_min_ = −0.29 e Å^−^^3^
357 parameters	Absolute structure: Flack (1983)
1 restraint	Absolute structure parameter: 0.4 (6)

### Special details

**Table:**

Refinement. X-ray diffraction data was collected on a Bruker SMART APEX2 CCD diffractometer installed at a rotating anode source (MoKα radiation, λ=0.71073 Å), and equipped with an Oxford Cryosystems (Cryostream700) nitrogen gas-flow apparatus. The data were collected by the rotation method with a 0.5° frame-width (ω scan) and a 15 second exposure per frame. Three sets of data (360 frames in each set) were collected, nominally covering complete reciprocal space. The structure was solved in the Olex2 (Dolomanov, O. V. B. *et al.*, 2009) crystallography program using the XS structure solution program (Sheldrick,G. M, 2008) using the Charge Flipping method and refined using the olex2.refine refinement package(Bourhis, L. J., *et al.*, 2015) using least-squares minimization.

### Fractional atomic coordinates and isotropic or equivalent isotropic displacement parameters (Å^2^)

**Table:**

	*x*	*y*	*z*	*U*_iso_*/*U*_eq_	Occ. (<1)
O1	0.49355 (18)	0.45455 (6)	0.44984 (6)	0.01210 (15)	
O2	0.4742 (2)	0.55289 (7)	0.55860 (6)	0.01868 (18)	
O3	0.8313 (2)	0.67789 (6)	0.46065 (6)	0.01575 (17)	
H3	0.921 (4)	0.6856 (13)	0.5086 (12)	0.014 (4)*	
O4	0.9691 (2)	0.55149 (6)	0.31883 (6)	0.01423 (16)	
H4	1.069 (6)	0.607 (2)	0.3253 (17)	0.0213 (2)*	0.75 (2)
O5	0.98812 (19)	0.35794 (6)	0.47231 (5)	0.01216 (15)	
H5	1.142 (5)	0.3877 (16)	0.4705 (14)	0.031 (6)*	
O6	0.8176 (2)	0.19330 (6)	0.38768 (6)	0.01444 (16)	
H6	0.716 (5)	0.1509 (15)	0.4013 (13)	0.026 (5)*	
C1	0.5661 (2)	0.53643 (8)	0.49141 (8)	0.01170 (19)	
C2	0.7521 (2)	0.58827 (7)	0.44491 (7)	0.01084 (18)	
C3	0.8080 (2)	0.53592 (7)	0.37784 (7)	0.00969 (18)	
C4	0.6454 (2)	0.44679 (7)	0.37658 (7)	0.00959 (17)	
H4a	0.5081 (2)	0.44220 (7)	0.32212 (7)	0.0115 (2)*	
C5	0.8294 (2)	0.35942 (8)	0.38832 (7)	0.00976 (17)	
H5a	0.9613 (2)	0.35849 (8)	0.34430 (7)	0.0117 (2)*	
C6	0.6423 (2)	0.27408 (8)	0.37799 (8)	0.01268 (19)	
H6a	0.5258 (2)	0.27417 (8)	0.32035 (8)	0.0152 (2)*	
H6b	0.5135 (2)	0.27418 (8)	0.42206 (8)	0.0152 (2)*	
O7	0.86622 (18)	0.29619 (5)	0.00750 (5)	0.00983 (14)	
O8	1.02947 (19)	0.17939 (6)	−0.06529 (6)	0.01324 (16)	
O9	0.6792 (2)	0.05632 (6)	0.03244 (6)	0.01390 (16)	
H9	0.558 (5)	0.0416 (17)	−0.0046 (16)	0.032 (6)*	
O10	0.39236 (18)	0.19546 (6)	0.13751 (6)	0.01330 (15)	
H10	0.353 (13)	0.1386 (9)	0.137 (4)	0.0199 (2)*	0.25 (2)
O11	1.0111 (2)	0.30023 (6)	0.20099 (5)	0.01293 (15)	
H11	1.115 (4)	0.2763 (15)	0.1732 (13)	0.026 (5)*	
O12	0.7556 (2)	0.51222 (7)	0.09005 (7)	0.01958 (19)	
H12	0.845 (5)	0.5638 (16)	0.0855 (14)	0.033 (6)*	
C7	0.8780 (2)	0.20343 (7)	−0.01219 (7)	0.00978 (18)	
C8	0.6981 (2)	0.15146 (7)	0.03486 (7)	0.00985 (18)	
C9	0.5682 (2)	0.21089 (7)	0.08490 (7)	0.00962 (18)	
C10	0.6682 (2)	0.30805 (7)	0.06851 (7)	0.00877 (17)	
H10a	0.5036 (2)	0.34608 (7)	0.04143 (7)	0.0105 (2)*	
C11	0.8137 (2)	0.35734 (8)	0.14937 (7)	0.00942 (17)	
H11a	0.6630 (2)	0.37406 (8)	0.18475 (7)	0.0113 (2)*	
C12	0.9591 (2)	0.44733 (8)	0.12926 (8)	0.01143 (18)	
H12a	1.0989 (2)	0.43428 (8)	0.08998 (8)	0.0137 (2)*	
H12b	1.0615 (2)	0.47396 (8)	0.18315 (8)	0.0137 (2)*	
N1	0.7273 (2)	0.19521 (7)	0.69016 (6)	0.01118 (16)	
H1	0.8437 (2)	0.15248 (7)	0.67582 (6)	0.0134 (2)*	0.25 (2)
N2	−0.1522 (2)	0.54416 (7)	0.82171 (6)	0.01059 (16)	
H2	−0.249 (5)	0.5896 (18)	0.8370 (15)	0.01271 (19)*	0.75 (2)
C13	0.5899 (2)	0.18296 (8)	0.75790 (8)	0.01196 (19)	
H13	0.6182 (2)	0.12658 (8)	0.78939 (8)	0.0144 (2)*	
C14	0.4091 (2)	0.24834 (8)	0.78414 (8)	0.01189 (19)	
H14	0.3166 (2)	0.23705 (8)	0.83281 (8)	0.0143 (2)*	
C15	0.3638 (2)	0.33182 (7)	0.73790 (7)	0.00892 (17)	
C16	0.5031 (2)	0.34329 (8)	0.66607 (7)	0.01098 (19)	
H16	0.4744 (2)	0.39804 (8)	0.63217 (7)	0.0132 (2)*	
C17	0.6837 (2)	0.27425 (8)	0.64452 (7)	0.01128 (18)	
H17	0.7792 (2)	0.28315 (8)	0.59609 (7)	0.0135 (2)*	
C18	−0.1299 (2)	0.46219 (8)	0.86276 (7)	0.01123 (19)	
H18	−0.2282 (2)	0.45253 (8)	0.91050 (7)	0.0135 (2)*	
C19	0.0339 (3)	0.39174 (8)	0.83650 (7)	0.01045 (18)	
H19	0.0474 (3)	0.33395 (8)	0.86598 (7)	0.0125 (2)*	
C20	0.1802 (2)	0.40554 (7)	0.76632 (7)	0.00874 (17)	
C21	0.1521 (3)	0.49220 (8)	0.72563 (8)	0.0149 (2)	
H21	0.2475 (3)	0.50417 (8)	0.67768 (8)	0.0179 (3)*	
C22	−0.0134 (3)	0.56029 (8)	0.75481 (8)	0.0146 (2)	
H22	−0.0293 (3)	0.61916 (8)	0.72725 (8)	0.0176 (3)*	

### Atomic displacement parameters (Å^2^)

**Table:**

	*U*^11^	*U*^22^	*U*^33^	*U*^12^	*U*^13^	*U*^23^
O1	0.0126 (4)	0.0088 (3)	0.0165 (4)	−0.0019 (3)	0.0075 (3)	0.0001 (3)
O2	0.0275 (5)	0.0128 (4)	0.0190 (4)	0.0043 (3)	0.0144 (4)	0.0019 (3)
O3	0.0253 (4)	0.0062 (3)	0.0153 (4)	−0.0035 (3)	0.0013 (3)	−0.0006 (3)
O4	0.0203 (4)	0.0101 (4)	0.0142 (4)	−0.0039 (3)	0.0089 (3)	0.0005 (3)
O5	0.0126 (3)	0.0114 (3)	0.0122 (3)	−0.0020 (3)	0.0007 (3)	0.0016 (3)
O6	0.0203 (4)	0.0068 (3)	0.0170 (4)	−0.0012 (3)	0.0053 (3)	0.0003 (3)
C1	0.0138 (5)	0.0078 (4)	0.0143 (5)	0.0021 (4)	0.0050 (4)	0.0008 (4)
C2	0.0145 (5)	0.0064 (4)	0.0121 (5)	−0.0009 (3)	0.0036 (4)	0.0011 (3)
C3	0.0114 (4)	0.0073 (4)	0.0106 (4)	−0.0012 (3)	0.0022 (3)	0.0015 (3)
C4	0.0103 (4)	0.0079 (4)	0.0109 (4)	−0.0006 (3)	0.0028 (3)	0.0006 (3)
C5	0.0125 (4)	0.0073 (4)	0.0098 (4)	−0.0011 (3)	0.0027 (3)	−0.0002 (3)
C6	0.0152 (5)	0.0073 (4)	0.0155 (5)	−0.0021 (4)	0.0021 (4)	0.0004 (4)
O7	0.0139 (3)	0.0068 (3)	0.0100 (3)	0.0000 (3)	0.0060 (3)	−0.0005 (3)
O8	0.0184 (4)	0.0109 (4)	0.0118 (4)	0.0030 (3)	0.0067 (3)	−0.0004 (3)
O9	0.0167 (4)	0.0056 (3)	0.0182 (4)	−0.0009 (3)	−0.0016 (3)	−0.0008 (3)
O10	0.0126 (3)	0.0093 (3)	0.0198 (4)	−0.0007 (3)	0.0086 (3)	0.0026 (3)
O11	0.0173 (4)	0.0122 (3)	0.0095 (3)	0.0056 (3)	0.0029 (3)	0.0017 (3)
O12	0.0169 (4)	0.0100 (4)	0.0295 (5)	−0.0025 (3)	−0.0044 (4)	0.0079 (4)
C7	0.0125 (4)	0.0077 (4)	0.0089 (4)	0.0009 (3)	0.0006 (3)	0.0000 (3)
C8	0.0115 (4)	0.0061 (4)	0.0120 (5)	−0.0003 (3)	0.0019 (4)	−0.0001 (3)
C9	0.0088 (4)	0.0078 (4)	0.0122 (4)	−0.0004 (3)	0.0015 (3)	0.0015 (3)
C10	0.0106 (4)	0.0063 (4)	0.0103 (4)	0.0008 (3)	0.0047 (3)	0.0010 (3)
C11	0.0123 (4)	0.0073 (4)	0.0093 (4)	0.0015 (3)	0.0037 (3)	0.0007 (3)
C12	0.0134 (5)	0.0081 (4)	0.0127 (4)	−0.0013 (4)	0.0016 (4)	0.0000 (4)
N1	0.0116 (4)	0.0101 (4)	0.0121 (4)	0.0017 (3)	0.0027 (3)	−0.0020 (3)
N2	0.0117 (4)	0.0083 (4)	0.0125 (4)	0.0025 (3)	0.0039 (3)	−0.0013 (3)
C13	0.0147 (5)	0.0086 (4)	0.0132 (5)	0.0026 (4)	0.0039 (4)	0.0005 (4)
C14	0.0144 (5)	0.0094 (4)	0.0130 (5)	0.0022 (4)	0.0059 (4)	0.0013 (4)
C15	0.0095 (4)	0.0073 (4)	0.0104 (4)	0.0006 (3)	0.0027 (3)	−0.0012 (3)
C16	0.0135 (5)	0.0097 (4)	0.0104 (4)	0.0014 (3)	0.0038 (4)	−0.0006 (3)
C17	0.0135 (4)	0.0106 (4)	0.0104 (4)	0.0015 (4)	0.0040 (3)	−0.0014 (4)
C18	0.0135 (5)	0.0102 (4)	0.0110 (5)	0.0003 (3)	0.0052 (4)	−0.0009 (3)
C19	0.0144 (5)	0.0083 (4)	0.0096 (4)	0.0008 (3)	0.0046 (4)	0.0000 (3)
C20	0.0100 (4)	0.0069 (4)	0.0098 (4)	0.0006 (3)	0.0034 (3)	−0.0014 (3)
C21	0.0204 (5)	0.0099 (5)	0.0171 (5)	0.0055 (4)	0.0118 (4)	0.0041 (4)
C22	0.0204 (5)	0.0093 (5)	0.0162 (5)	0.0051 (4)	0.0092 (4)	0.0038 (4)

### Geometric parameters (Å, º)

**Table:**

O1—C1	1.3677 (14)	C8—C9	1.3694 (16)
O1—C4	1.4494 (13)	C9—C10	1.5130 (15)
O2—C1	1.2223 (15)	C10—H10a	1.0000
O3—H3	0.816 (19)	C10—C11	1.5290 (16)
O3—C2	1.3579 (14)	C11—H11a	1.0000
O4—H4	0.93 (3)	C11—C12	1.5251 (16)
O4—C3	1.3062 (14)	C12—H12a	0.9900
O5—H5	0.86 (2)	C12—H12b	0.9900
O5—C5	1.4207 (14)	N1—H1	0.8800
O6—H6	0.83 (2)	N1—C13	1.3394 (15)
O6—C6	1.4283 (15)	N1—C17	1.3449 (15)
C1—C2	1.4376 (16)	N2—H2	0.85 (3)
C2—C3	1.3523 (16)	N2—C18	1.3419 (15)
C3—C4	1.4991 (15)	N2—C22	1.3403 (15)
C4—H4a	1.0000	C13—H13	0.9500
C4—C5	1.5306 (16)	C13—C14	1.3802 (16)
C5—H5a	1.0000	C14—H14	0.9500
C5—C6	1.5142 (16)	C14—C15	1.4049 (16)
C6—H6a	0.9900	C15—C16	1.3992 (15)
C6—H6b	0.9900	C15—C20	1.4858 (14)
O7—C7	1.3746 (13)	C16—H16	0.9500
O7—C10	1.4499 (13)	C16—C17	1.3897 (15)
O8—C7	1.2296 (14)	C17—H17	0.9500
O9—H9	0.79 (3)	C18—H18	0.9500
O9—C8	1.3739 (13)	C18—C19	1.3803 (16)
O10—H10	0.8400	C19—H19	0.9500
O10—C9	1.2794 (14)	C19—C20	1.4012 (15)
O11—H11	0.79 (2)	C20—C21	1.3998 (15)
O11—C11	1.4134 (14)	C21—H21	0.9500
O12—H12	0.86 (2)	C21—C22	1.3789 (16)
O12—C12	1.4217 (15)	C22—H22	0.9500
C7—C8	1.4240 (16)		
			
C4—O1—C1	108.88 (8)	C11—C10—O7	109.96 (9)
C2—O3—H3	113.0 (13)	C11—C10—C9	113.89 (9)
C5—O5—H5	107.8 (15)	C11—C10—H10a	109.35 (6)
C6—O6—H6	105.8 (15)	C10—C11—O11	112.89 (9)
O2—C1—O1	118.74 (10)	H11a—C11—O11	107.21 (5)
C2—C1—O1	109.77 (10)	H11a—C11—C10	107.21 (6)
C2—C1—O2	131.48 (11)	C12—C11—O11	109.14 (9)
C1—C2—O3	125.30 (11)	C12—C11—C10	112.86 (9)
C3—C2—O3	126.19 (10)	C12—C11—H11a	107.21 (6)
C3—C2—C1	108.15 (10)	C11—C12—O12	110.23 (9)
C2—C3—O4	131.22 (10)	H12a—C12—O12	109.61 (7)
C4—C3—O4	119.67 (10)	H12a—C12—C11	109.61 (6)
C4—C3—C2	109.11 (10)	H12b—C12—O12	109.61 (6)
C3—C4—O1	103.96 (9)	H12b—C12—C11	109.61 (6)
H4a—C4—O1	109.96 (6)	H12b—C12—H12a	108.1
H4a—C4—C3	109.96 (6)	C17—N1—C13	118.57 (10)
C5—C4—O1	108.21 (9)	C22—N2—C18	120.98 (10)
C5—C4—C3	114.57 (9)	H13—C13—N1	118.44 (6)
C5—C4—H4a	109.96 (6)	C14—C13—N1	123.12 (11)
C4—C5—O5	110.05 (9)	C14—C13—H13	118.44 (7)
H5a—C5—O5	109.63 (6)	H14—C14—C13	120.45 (7)
H5a—C5—C4	109.63 (6)	C15—C14—C13	119.10 (10)
C6—C5—O5	108.26 (9)	C15—C14—H14	120.45 (6)
C6—C5—C4	109.62 (9)	C16—C15—C14	117.47 (10)
C6—C5—H5a	109.63 (6)	C20—C15—C14	120.67 (9)
C5—C6—O6	108.86 (9)	C20—C15—C16	121.84 (9)
H6a—C6—O6	109.91 (6)	H16—C16—C15	120.14 (6)
H6a—C6—C5	109.91 (6)	C17—C16—C15	119.73 (10)
H6b—C6—O6	109.91 (6)	C17—C16—H16	120.14 (7)
H6b—C6—C5	109.91 (6)	C16—C17—N1	122.00 (10)
H6b—C6—H6a	108.3	H17—C17—N1	119.00 (6)
C10—O7—C7	108.36 (8)	H17—C17—C16	119.00 (7)
C8—O9—H9	109.1 (18)	H18—C18—N2	119.58 (6)
C11—O11—H11	111.1 (15)	C19—C18—N2	120.83 (10)
C12—O12—H12	106.7 (15)	C19—C18—H18	119.58 (7)
O8—C7—O7	118.26 (10)	H19—C19—C18	120.03 (7)
C8—C7—O7	110.31 (9)	C20—C19—C18	119.94 (10)
C8—C7—O8	131.42 (10)	C20—C19—H19	120.03 (6)
C7—C8—O9	123.53 (10)	C19—C20—C15	121.17 (9)
C9—C8—O9	127.32 (10)	C21—C20—C15	121.48 (9)
C9—C8—C7	109.03 (10)	C21—C20—C19	117.33 (10)
C8—C9—O10	130.96 (10)	H21—C21—C20	119.85 (6)
C10—C9—O10	121.55 (10)	C22—C21—C20	120.30 (11)
C10—C9—C8	107.50 (9)	C22—C21—H21	119.85 (7)
C9—C10—O7	104.79 (8)	C21—C22—N2	120.61 (11)
H10a—C10—O7	109.35 (5)	H22—C22—N2	119.70 (6)
H10a—C10—C9	109.35 (6)	H22—C22—C21	119.70 (7)

### Hydrogen-bond geometry (Å, º)

**Table:**

*D*—H···*A*	*D*—H	H···*A*	*D*···*A*	*D*—H···*A*
N2—H2···O10^i^	0.86 (3)	1.74 (3)	2.5862 (14)	169 (2)
O3—H3···O5^ii^	0.817 (19)	2.531 (19)	2.8832 (13)	107.5 (15)
O3—H3···O6^ii^	0.817 (19)	1.903 (19)	2.7117 (14)	170.0 (19)
O4—H4···N1^ii^	0.93 (3)	1.64 (3)	2.5428 (14)	163 (3)
O5—H5···O1^iii^	0.85 (2)	2.00 (2)	2.8510 (13)	173 (2)
O6—H6···O2^iv^	0.83 (2)	1.84 (2)	2.6616 (14)	173 (2)
O9—H9···O12^v^	0.79 (2)	1.91 (2)	2.6902 (14)	175 (2)
O11—H11···O10^iii^	0.79 (2)	1.91 (2)	2.6663 (13)	162 (2)
O12—H12···O8^vi^	0.87 (2)	1.81 (2)	2.6683 (14)	169 (2)
C5—H5A···O11	1.00 (1)	2.44 (1)	3.2950 (14)	143 (1)
C12—H12B···O4	0.99 (1)	2.50 (1)	3.3249 (16)	141 (1)
C14—H14···O8^vii^	0.95 (1)	2.40 (1)	3.3311 (15)	166 (1)
C16—H16···O2	0.95 (1)	2.51 (1)	3.4513 (16)	170 (1)
C17—H17···O5	0.95 (1)	2.55 (1)	3.4651 (14)	163 (1)
C19—H19···O7^vii^	0.95 (1)	2.56 (1)	3.2181 (14)	127 (1)
C19—H19···O8^vii^	0.95 (1)	2.48 (1)	3.4267 (15)	174 (1)
C21—H21···O2	0.95 (1)	2.40 (1)	3.3418 (17)	173 (1)
C22—H22···O6^viii^	0.95 (1)	2.44 (1)	3.1860 (16)	136 (1)

Symmetry codes: (i) −*x*, *y*+1/2, −*z*+1; (ii) −*x*+2, *y*+1/2, −*z*+1; (iii) *x*+1, *y*, *z*; (iv) −*x*+1, *y*−1/2, −*z*+1; (v) −*x*+1, *y*−1/2, −*z*; (vi) −*x*+2, *y*+1/2, −*z*; (vii) *x*−1, *y*, *z*+1; (viii) −*x*+1, *y*+1/2, −*z*+1.
